# Federated-learning-based prognosis assessment model for acute pulmonary thromboembolism

**DOI:** 10.1186/s12911-024-02543-x

**Published:** 2024-05-27

**Authors:** Jun Zhou, Xin Wang, Yiyao Li, Yuqing Yang, Juhong Shi

**Affiliations:** 1 State Key Laboratory of Networking and Switching Technology, Beijing University of Posts and Telecommunications, Beijing, China; 2 Department of Ultrasound, Peking Union Medical College Hospital, Beijing, China; 3 Department of Pulmonary and Critical Care Medicine, Peking Union Medical College Hospital, Beijing, China

**Keywords:** Acute pulmonary thromboembolism, Federated learning, Prognosis assessment model, Patient data privacy, Machine learning

## Abstract

**Background:**

Acute pulmonary thromboembolism (PTE) is a common cardiovascular disease and recognizing low prognosis risk patients with PTE accurately is significant for clinical treatment. This study evaluated the value of federated learning (FL) technology in PTE prognosis risk assessment while ensuring the security of clinical data.

**Methods:**

A retrospective dataset consisted of PTE patients from 12 hospitals were collected, and 19 physical indicators of patients were included to train the FL-based prognosis assessment model to predict the 30-day death event.

Firstly, multiple machine learning methods based on FL were compared to choose the superior model. And then performance of models trained on the independent (IID) and non-independent identical distributed(Non-IID) datasets was calculated and they were tested further on Real-world data. Besides, the optimal model was compared with pulmonary embolism severity index (PESI), simplified PESI (sPESI), Peking Union Medical College Hospital (PUMCH).

**Results:**

The area under the receiver operating characteristic curve (AUC) of logistic regression(0.842) outperformed convolutional neural network (0.819) and multi layer perceptron (0.784). Under IID, AUC of model trained using FL(Fed) on the training, validation and test sets was 0.852 ± 0.002, 0.867 ± 0.012 and 0.829 ± 0.004. Under Real-world, AUC of Fed was 0.855 ± 0.005, 0.882 ± 0.003 and 0.835 ± 0.005. Under IID and Real-world, AUC of Fed surpassed centralization model(NonFed) (0.847 ± 0.001, 0.841 ± 0.001 and 0.811 ± 0.001). Under Non-IID, although AUC of Fed (0.846 ± 0.047) outperformed NonFed (0.841 ± 0.001) on validation set, it (0.821 ± 0.016 and 0.799 ± 0.031) slightly lagged behind NonFed (0.847 ± 0.001 and 0.811 ± 0.001) on the training and test sets.

In practice, AUC of Fed (0.853, 0.884 and 0.842) outshone PESI (0.812, 0.789 and 0.791), sPESI (0.817, 0.770 and 0.786) and PUMCH(0.848, 0.814 and 0.832) on the training, validation and test sets. Additionally, Fed (0.842) exhibited higher AUC values across test sets compared to those trained directly on the clients (0.758, 0.801, 0.783, 0.741, 0.788).

**Conclusions:**

In this study, the FL based machine learning model demonstrated commendable efficacy on PTE prognostic risk prediction, rendering it well-suited for deployment in hospitals.

## Background

Pulmonary thromboembolism (PTE) refers to the disease caused by the obstruction of the pulmonary artery or its branches due to thrombi originating from the venous system or right heart. It is a common and life-threatening cardiovascular disease, causing 60,000 to 100,000 deaths annually. [1, 2] With advances in diagnostic techniques, the incidence rate of PTE increased notably in recent years, especially among the elderly patients [1, 3]. Due to its increased incidence, high risk of death, and substantial socioeconomic burden, the assessment and stratification of the prognosis risk in PTE patients are crucial and mandatory, providing basis for further disease management and treatment. For instance, patients with low risk could be discharged early or receive outpatient management with the use of anticoagulant medications such as heparin and warfarin, whereas patients with high risk may derive greater benefits from thrombolytic therapy, interventional treatment and a more intensive surveillance in an intensive care setting [4]. Furthermore, the assessment of prognosis in PTE requires a comprehensive consideration of clinical symptoms, radiological features, laboratory test parameters, and the presence of comorbidities and complications associated with the severity of PTE [5]. Currently, several different models demonstrating varying performance are available in clinical practice to help identify PTE patients with low prognostic risk, such as the pulmonary embolism severity index (PESI), or its simplified version, the simplified pulmonary embolism severity index (sPESI), which are recommended by guidelines of the European Cardiology Society and Respiratory Society, and a recently proposed model, the Peking Union Medical College Hospital (PUMCH) rule, which showed higher discriminative ability for predicting 30-day probability of PTE deaths in a Chinese dataset (Table 1) [5–8]. Nevertheless, research on advance of PTE prognosis model performance is still in need to reduce serious disabilities, deaths, and economic losses.

**Table 1:** Characteristics and weights of PESI, sPESI, and PUMCH scoring models

Variable	PESI	sPESI	PUMCH rule
Age	Age in years	1 point (if age > 80 years)	2 points (if age > 80 years)
Male sex	10 points		1 point
Cancer	30 points	1 point	5 points
Chronic heart failure	10 points	1 point	2 points
Chronic pulmonary disease	10 points		3 points
Pulse rate ≥110 bpm	20 points	1 point	3 points
Systolic blood pressure < 100 mm Hg	30 points	1 point	3 points
Respiratory rate > 30 breaths per minute	20 points		
Temperature < 36 °C	20 points		
Altered mental status	60 points		2 points
Arterial oxyhaemoglobin saturation < 90%	20 points	1 point	1 point
Serum calcium ≤ 2.13 mmol/L			1 point

In the real world, when constructing a prognostic model for PTE based on patient data, the data from individual hospitals is typically limited, making it difficult to obtain sufficient data in one hospital to train artificial intelligence (AI), especially the deep learning models. Moreover, the patients distribution of one hospital usually has its own disease spectrum, which may differ greatly from the data distribution of other hospitals. Recently, it has been proposed that training models on such biased datasets that cannot represent real-world clinical or patient diversity (such as a dataset from a certain hospital) will bring some limitations to the application of AI in the medical field [9–11]. Compared to training AI models using larger (less diverse) datasets from a single center, using data from multiple centers has shown greater potential to train more accurate and generalizable AI models [11–17].

During the process of aggregating data from multiple centers to build a model, the traditional multicenter modeling process involves transferring data from multiple centers to a single hospital for processing, merging, and modeling. However, this approach entails the risk of data leakage, leading to data security concerns and compromising patient privacy [18]. Most hospitals are reluctant to transmit raw data over the network to external sources especially when there are risks in their information systems. Additionally, legal and regulatory barriers increasingly restrict dataset aggregation for AI model training to protect data privacy and prevent data from being transferred outside the region of origin [19, 20]. Therefore, in the process of constructing prognostic models for PTE based on multicenter data, it is necessary to employ more secure and robust methods for model development.

Distributed learning allows AI models to be trained on multiple edge devices without the need for data to leave their original positions [21, 22]. In 2017, Google proposed Federated Learning (FL) framework [23], which is divided into two parts: client and server. Assuming the client is a hospital, FL allows hospitals to save data locally without uploading local data to the server. The server will issue a model to the hospital, and the hospital only needs to upload the parameters of the model trained with local data to the server to benefit from it. This not only meets the requirement that hospitals do not want to share patient data, but also protects the personal privacy of patients.

In the past few years, FL has been widely adopted in medicine to build computer aided diagnosis model while ensuring the data security. For example,Jean Ogier du Terrail et al. use FL to predict histological response to neoadjuvant chemotherapy in triple-negative breast cancer. They show that collaborative training of AI models further improves performance, on par with the best current approaches in which AI models are trained using time-consuming expert annotations [24]. Ittai Dayan et al. apply FL to predict clinical outcomes in patients with COVID-19 and the FL model provides 16% improvement in average AUC measured across all participating sites and an average increase in generalizability of 38% when compared with models trained at a single site using that site’s data [25]. Sarthak Pati et al. develop an accurate and generalizable machine learning model using FL for detecting glioblastoma sub-compartment boundaries. They demonstrate a 33% delineation improvement for the surgically targetable tumor and 23% for the complete tumor extent, over a publicly trained model [26]. Arash Heidari et al. use FL to develop a new lung cancer detection method based on the chest CT images. The findings show that the technique delivers 99.69% accuracy with the smallest possible categorization error [27]. In summary, the widespread adoption of FL in medicine has yielded significant achievements, providing an effective means for constructing high-performance AI models.

**Fig. 1:**
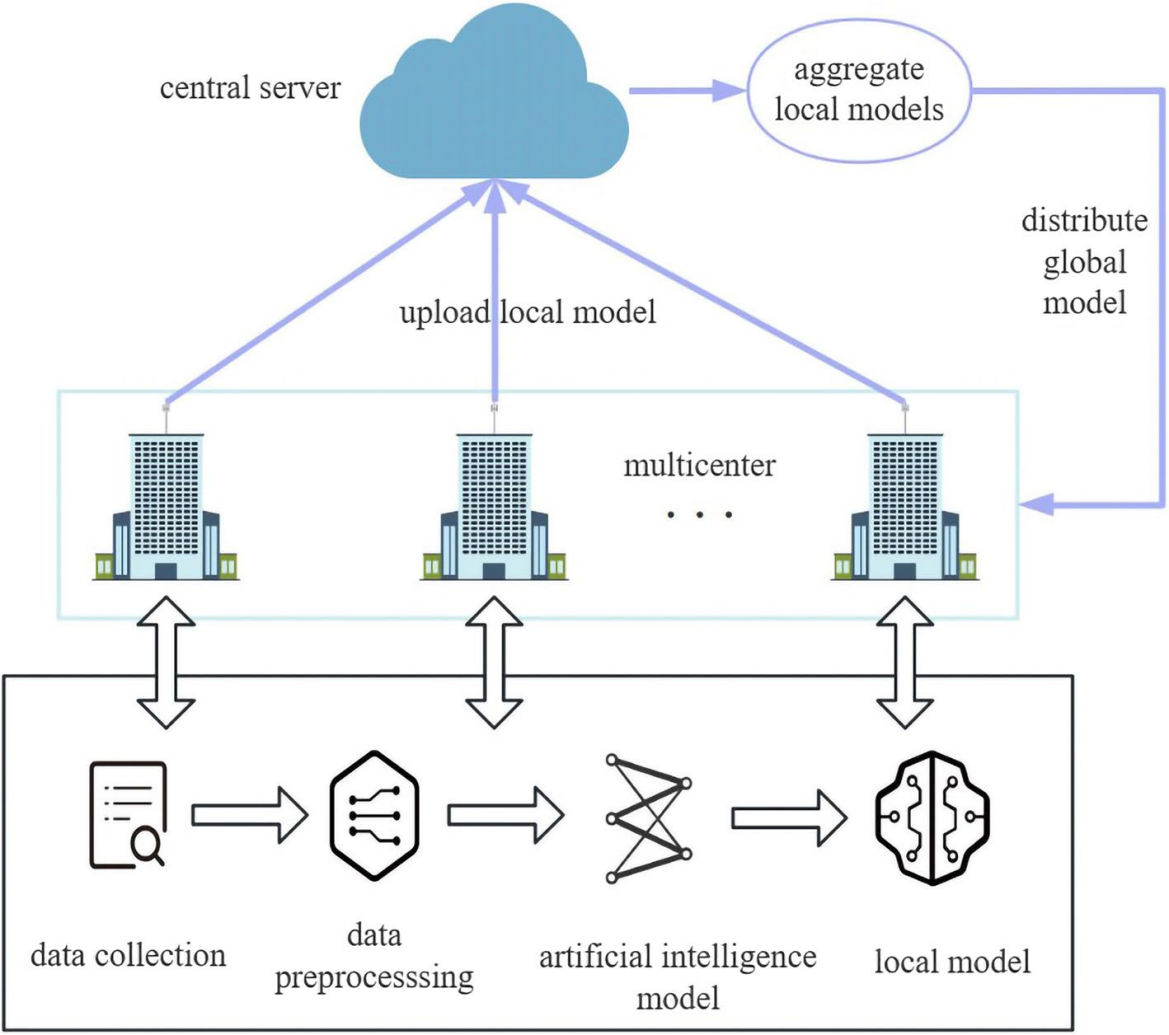
Federated learning process diagram

Currently, there is no existing research on FL specific to PTE prognostic risk. In this paper, we explore the application of FL technology for constructing prognostic models for PTE on a multicenter dataset, assessing the feasibility of FL for assisting in PTE prognosis risk prediction (Fig. 1). A detailed comparison of the impact of FL under independent and non-independent identically distributed scenarios is provided, and its performance is validated on real clinical data. Furthermore, a comparison is made with multiple clinically established PTE scoring models (PESI, sPESI, PUMCH).

## Methods

### Datasets

Patients from 12 hospitals in China aged ≥ 18 who were diagnosed with PTE between February 2010 and June 2020. Patients from other healthcare facilities were removed due to incomplete medical records. We only considered the first available medical record of patients with repeated hospitalizations due to PTE. All patients completed at least a 1-month follow-up and confirmed their survival status as of July 5, 2020. The detailed enrollment criterions could be found in previous study [8].

Patients’ clinical variables, including demographic information, medical history, related risk factors, and 30-day PTE all-cause mortality were primarily collected and reviewed by three doctors. The variables utilized in three PTE prognosis prediction rules, PESI, sPESI, and the PUMCH, (age, sex, body temperature, pulse rate, respiratory rate, blood pressure[BP], cancer, chronic heart failure, chronic pulmonary disease, altered mental status, arterial oxyhaemoglobin saturation and serum calcium level) were collected.

Considering that some of the clinical variables in the collected data had both continuous data and corresponding discrete data, such as age and age greater than 80, temperature and temperature lower than 36, etc. So a comparison experiment was conducted to investigate whether it was possible to use only discrete or continuous data of these clinical variables.

This multicenter observational study was approved by the Institutional Review Board of the Peking Union Medical College Hospital (PUMCH) (Ethical review number: I-22PJ1055) according to the Declaration of Helsinki.

### Data preprocessing

Since the collected data was not complete and there were missing values, before using the data to train the model, the mean value corresponding to each feature column was used to fill in the positions where the feature column had missing values. Specifically, when dealing with missing body temperature values, we calculated the average temperature of patients with recorded temperature data. This involved summing up all available temperature readings and dividing by the total number of patients with temperature data. Subsequently, we assigned this calculated average temperature as the value for patients with missing temperature data. We followed a similar approach for other features with missing values. After that, considering the inconsistency of the values corresponding to each feature column, for example, some feature columns had values below 10 while others had values above 100, in order to make each feature column in the same range, standardization was employed. Standardization involves transforming the features of a dataset onto a common scale, typically with a mean of 0 and a standard deviation of 1. The process of sample standardization typically involves the following steps:

- Compute the mean of each feature: For each feature, calculate the average of all sample values.

1 $$\mu =\frac{1}{n}{\sum }_{i=1}^{n}{x}_{i}$$

b. Compute the standard deviation of each feature: For each feature, calculate the standard deviation of all sample values.

2 $$\sigma =\sqrt{\frac{1}{n}{\sum }_{i=1}^{n}{\left({x}_{i}-\mu \right)}^{2}}$$

c. Standardize all sample values for each feature: Subtract the mean of the feature from each sample value, and then divide by the standard deviation of the feature.

3 $${x}_{standardize}=\frac{x-\mu }{\sigma }$$

### Dataset construction

Firstly, after filling in missing values and standardizing the data, several hospitals with relatively limited data volume were selected as the test set. Then, from the remaining data, 10% of the data from each hospital was randomly selected and summarized as the validation set. Finally, all remaining data was used as the training set.

In order to analyze the effect of FL under different data distributions, the experiment allocated the training set to clients according to the following three data distributions:

1. Independent Identically Distributed (IID). To simulate independent identically distribution of data between clients, positive and negative samples in the training set were assigned to clients in equal proportions, i.e., the sample labels 0 and 1 were the same in the data assigned to each client.
2. Non-Independent Identically Distributed (Non-IID). The positive and negative samples in the training set were distributed to the clients in different proportions, which made the amount of data and the proportion of 0 and 1 in the sample labels vary greatly between clients when constructing the dataset.
3. Real-world. Allocating the training set to the clients according to different hospital sources ensured that the datasets of the clients were from different hospitals.

### Statistical analysis

When analyzing models without multiple sets of experimental data, DeLong test was used to assess the significance of differences among various models. For models with multiple experiments, exemplified by Model A and Model B, two-tailed t-test was employed to compare the results obtained from multiple experiments of Model A with those of Model B. This approach facilitated a more comprehensive analysis of the disparities between the data derived from multiple experiments of Model A and Model B.

### AI model selection

**Fig. 2:**
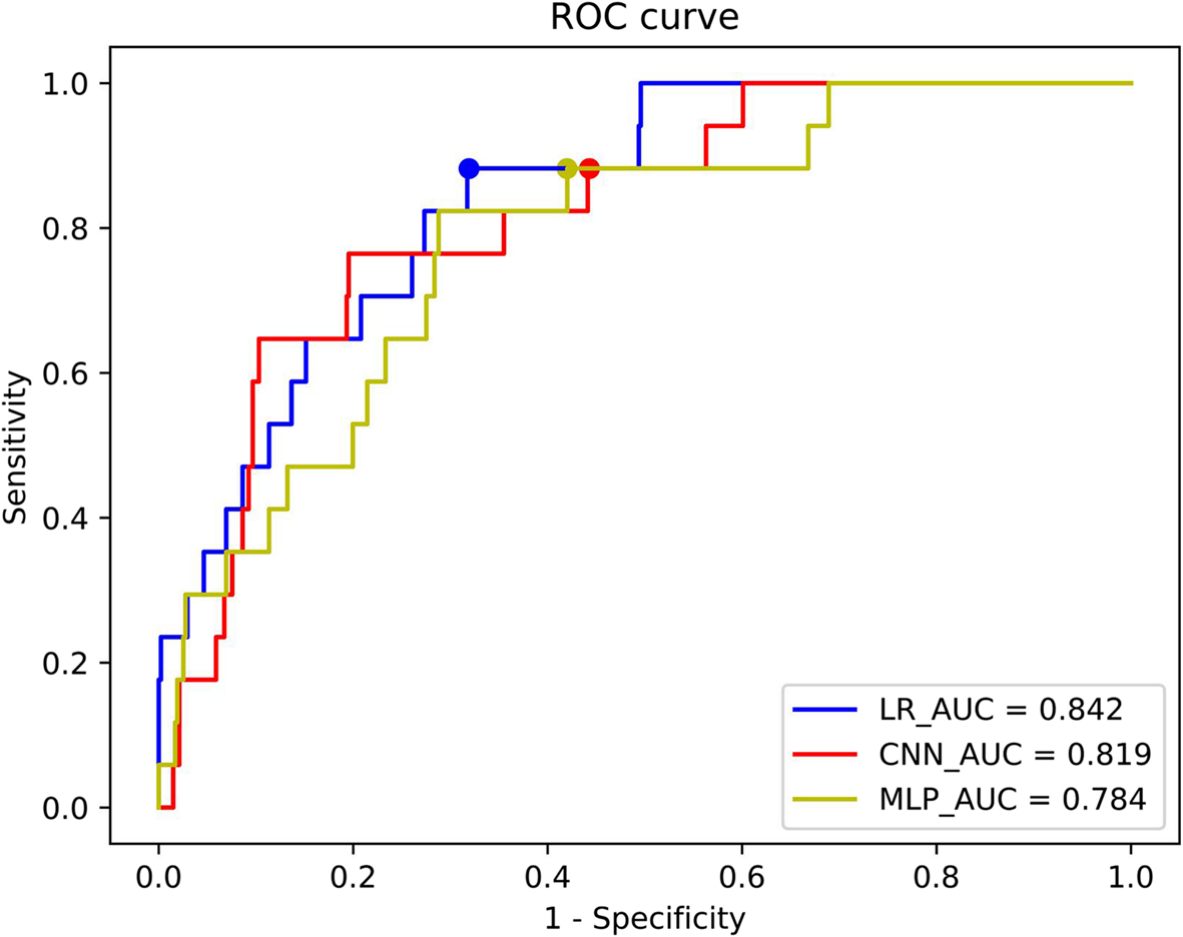
Receiver operating characteristic (ROC) curves of the three models trained using FL on the test set, the points marked in three colors in the figure are the corresponding classification thresholds of the three models

When simulating the experiments of FL, three models were first compared: the logistic regression (LR) model implemented using linear layers plus activation functions, convolutional neural network (CNN), and multi layer perceptron (MLP) model. In this experiment, we used real-world data, where the total dataset was divided among different clients based on the source hospital. As shown in Fig. 2, it was found that at the classification threshold corresponding to the model, although the sensitivity of the three models was the same (0.882), the specificity of LR (0.681) was higher than that of CNN (0.557) and MLP (0.580). And the overall area under the receiver operating characteristic (ROC) curve (AUC) value of LR (0.842) was higher than CNN (0.819) and MLP (0.784).

Delong tests were conducted to assess the significance of the trained models' performance. Models were compared against LR, which exhibited the highest AUC. This comparison was made using prediction results from the test set. The results indicated significant differences with MLP (*p* = 0.006 < 0.05) models, while CNN showed no significant differences (*p* = 0.530). However, LR(0.326) demonstrated a higher area under the precision-recall curve (AUPRC) compared to CNN (0.132). This suggested LR's superior performance in capturing the trade-off between precision and recall. Consequently, LR was selected as the model for the FL experiments.

Meanwhile, the performance of models trained using FL and centralization model were compared in the experiment. Centralization model refered to training the LR model directly on the training set, observing its impact on the validation set, and finally testing it on the test set. In addition to this, in order to compare with the scoring models that are often used in medicine, three models were selected: PESI, sPESI and PUMCH.

### FL process

The feasibility of deploying FL in five hospitals was experimentally studied, which protected data privacy during model training. In this FL scheme, the central server first initializes a global model randomly and then distribute it to five hospitals. Then, during each round of FL model updates, the central server first aggregates all local models and then uses the Federated Averaging (FedAvg) Algorithm [23] to update the global model parameters. The updated model parameters are generated by weighted averaging the parameters of all local models, which are proportional to the size of the local data provided by the hospital to the central server. Next the central server distributes the updated global model to each hospital, and then each hospital continues to make local updates based on the updated global model and its local data. After a round, each hospital sends the updated parameters to the central server for the next round of updates. This process is repeated until the global model converges. The detailed process of FedAvg is shown in Algorithm 1.

**Figure:**
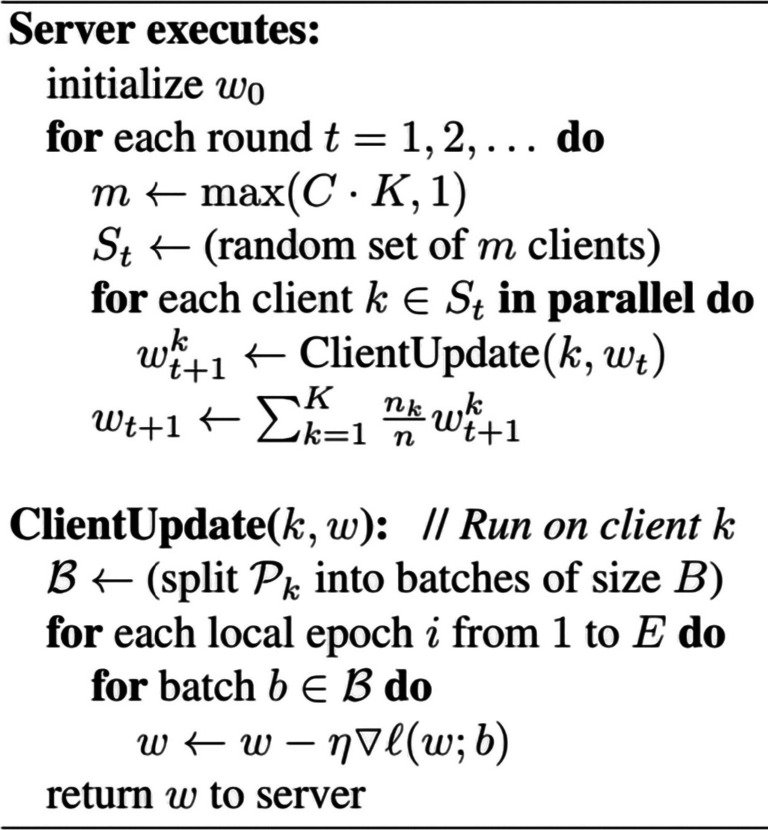
** Algorithm 1.** Federated Averaging. The K clients ar indexed by; B is the local minibatch size, E is the number of local epochs and η is the learning rate.

Formally, suppose that there are *K* (*K*=*5* in the experiment) hospitals performing the above FL scheme, where *n*_*k*_ is the amount of data for client *k*. In the *t*-*th* round of federated training, the central server firstly sends the global model parameters ω_t_ to each hospital, and then each hospital optimizes the received model locally with its own dataset for *E* epochs, and then uploads the updated local model parameters ω to the server. Once the updates of all hospitals' local models have been accepted, the server assigns weights to the local model parameters of hospital *k* based on the value of *n*_*k*_/*n* during server aggregation, where *n* is the total data volume of all hospitals. The process of these is repeated until the global model converges. Note that in this scenario, each hospital needs to upload the sample size of its local dataset to the server for weighted aggregation of the model on the server side.

The FedML [28] platform is used in the experiment to simulate FL, which is a research-oriented FL library and benchmark. FedML promotes diverse algorithmic research with flexible and generic API design and comprehensive reference baseline implementations [28]. With FedML, developers can easily deploy FL code to both the client and server side, making it easier to implement FL process.

### Model evaluation

During the experiment, data was distributed to five clients according to the three distributions mentioned above. And ten random experiments were conducted under each distribution. The accuracy, precision, recall, F1-score and AUC values of the model were recorded in ten experiments, and their mean and standard deviation were calculated as the final evaluation indicators for the model. It should be noted that considering the significant difference in the number of positive and negative samples in the PTE dataset, in order to improve the accuracy of the model in predicting positive samples, the classification threshold of the model was set to 0.3.

## Results

### Baseline information

The data used in the experiment came from a total of 3997 patients. The data was obtained from 12 hospitals and contained 19 physical indicators of the patients and the survival status of the patients during the 30 days of follow-up. A total of 176 patients died during the 30 days of follow-up, resulting in a mortality rate of 4.40% (176/3997). Specific information on the data is shown in Table 2.

**Table 2:** Patients demographic and clinical characteristics on the collected data

Variable name	All patients (*n* = 3997)	Missing value ratio
Age	68	
Age > 80	15.01% (600/3997)	
Male sex	49.91% (1995/3997)	
Altered mental status	13.31% (532/3997)	
Chronic heart failure	13.5% (473/3504)	12.33% (493/3997)
Chronic pulmonary disease	14.19% (567/3997)	
Cancer	15.44% (617/3997)	
Systolic BP (mm Hg)	121	0.7% (29/3997)
Systolic BP < 100 mm Hg	12.56% (502/3997)	
Pulse rate (bpm)	82	0.5% (20/3997)
Pulse rate ≥ 110 bpm	11.36% (454/3997)	
Temperature(°C)	36.5	1% (40/3997)
Temperature < 36 °C	1.6% (64/3997)	
Respiratory rate	19	1.45% (58/3997)
Respiratory rate > 30	2.33% (93/3997)	
Serum calcium(mmol/L)	2.2	1.5% (60/3997)
Serum calcium ≤ 2.13	32.15% (1285/3997)	
Arterial oxyhaemoglobin saturation	95	16.11% (644/3997)
Arterial oxyhaemoglobin Saturation < 90	13.71% (548/3997)	

*Abbreviation*: *BP* blood pressure

**Fig. 3:**
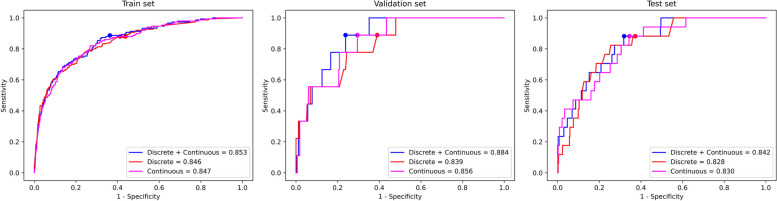
The results of the comparison experiments on the three groups, where the red curve corresponds to the experimental results on the group A, and the magenta curve and blue curve correspond to the experimental results on the group B and C, respectively. The points marked with three colors in the figure are the corresponding classification thresholds of the models trained on the three groups

Among the 19 physical indicators, some had both discrete and continuous values. Therefore, in the comparative experiment, all data were divided into three groups: group A contained only discrete data for these indicators; group B contained only continuous data for these indicators; and group C contained both discrete and continuous data for these indicators. The experimental results are shown in Fig. 3.

On the training, validation and test sets, AUC of model trained using group C(0.853, 0.884 and 0.842) are superior to that of model trained using group A (0.846, 0.839 and 0.828) and B (0.847, 0.856 and 0.830). Therefore, in subsequent experiments, both continuous data and corresponding discrete data were used simultaneously.

### Model assessment

**Fig. 4:**
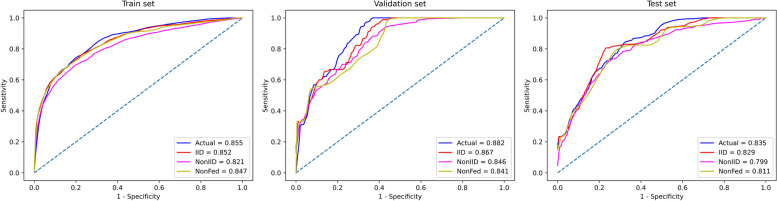
The ROC curves of the models trained using FL and centralization model on the training, validation and test sets. The curve in the figure is the average result of the curves obtained from ten experiments. The blue curve in the figure corresponds to the ROC curve of model trained using FL in Real-world, the red curve corresponds to IID, the magenta curve corresponds to Non-IID and the yellow curve corresponds to centralization model

In the experiment, three scenarios of FL were simulated: IID, Non-IID, and Real-world, as well as centralization model. The results of the experiment are shown in Fig. 4, and the specific values obtained from the experiment are recorded in Table 3.

**Table 3:** Models trained using FL and centralization model under three distributions

Characteristic	Actual	IID	NonIID	NonFed
Train set				
AUC	0.855 ± 0.005	0.852 ± 0.002	0.821 ± 0.016	0.847 ± 0.001
AUPRC	0.316 ± 0.015	0.395 ± 0.009	0.327 ± 0.027	0.382 ± 0.002
Accuracy	0.953 ± 0.001	0.957 ± 0.001	0.886 ± 0.157	0.839 ± 0.034
Precision	0.522 ± 0.030	0.587 ± 0.023	0.366 ± 0.152	0.188 ± 0.037
Recall	0.117 ± 0.019	0.327 ± 0.020	0.435 ± 0.204	0.679 ± 0.044
F1-score	0.191 ± 0.024	0.419 ± 0.016	0.339 ± 0.082	0.292 ± 0.037
Validation set				
AUC	0.882 ± 0.003	0.867 ± 0.012	0.846 ± 0.047	0.841 ± 0.001
AUPRC	0.204 ± 0.032	0.355 ± 0.038	0.296 ± 0.096	0.294 ± 0.029
Accuracy	0.969 ± 0.003	0.965 ± 0.004	0.882 ± 0.172	0.821 ± 0.040
Precision	0.283 ± 0.137	0.333 ± 0.046	0.192 ± 0.096	0.089 ± 0.023
Recall	0.133 ± 0.070	0.333 ± 0	0.455 ± 0.225	0.600 ± 0.057
F1-score	0.201 ± 0.070	0.337 ± 0.022	0.240 ± 0.089	0.154 ± 0.032
Test set				
AUC	0.835 ± 0.005	0.829 ± 0.004	0.799 ± 0.031	0.811 ± 0.001
AUPRC	0.311 ± 0.011	0.323 ± 0.010	0.226 ± 0.071	0.291 ± 0.002
Accuracy	0.970 ± 0.002	0.961 ± 0.003	0.876 ± 0.162	0.810 ± 0.040
Precision	0.792 ± 0.158	0.413 ± 0.065	0.175 ± 0.120	0.109 ± 0.014
Recall	0.176 ± 0.028	0.235 ± 0	0.312 ± 0.260	0.606 ± 0.092
F1-score	0.287 ± 0.040	0.298 ± 0.017	0.178 ± 0.079	0.183 ± 0.014
*p*-value	*p* < 0.001	*p* < 0.001	*p* = 0.242	––––

In the table, Actual corresponds to model trained using FL in Real-world, IID corresponds to model trained using FL under IID, NonIID corresponds to model trained using FL under Non-IID and NonFed corresponds to centralization model. Two-tailed T-tests were conducted to assess the significance of the FL models' performance compared to NonFed, based on predictions from the test set. The specific values in the table are composed of mean and standard deviation, which are calculated from the results of ten experiments

*Abbreviations*:* AUC* Area under the curve, *AUPRC* Area under the precision-recall curve

On the training set, AUC of model trained using FL in the context of IID (0.852 ± 0.002) and Real-world (0.855 ± 0.005) surpass AUC of centralization model (0.847 ± 0.001). In the scenario of Non-IID, AUC of model trained using FL (0.821 ± 0.016) slightly lags behind that of centralization model.

On the validation set, for the IID, Non-IID and Real-world settings, AUC of model trained using FL (0.867 ± 0.012, 0.846 ± 0.047 and 0.882 ± 0.003, respectively) outperform the centralization model (0.841 ± 0.001). On the test set, in both IID and Real-world scenarios, AUC of model trained using FL (0.829 ± 0.004, *p* < 0.001 and 0.835 ± 0.005, *p* < 0.001) are superior to centralization model (0.811 ± 0.001). In the scenario of Non-IID, AUC of model trained using FL (0.799 ± 0.031, *p* = 0.242) shows a slight reduction compared to centralization model.

Analysis of Table 3 reveals that across the test set, when compared to the centralization model (0.291 ± 0.002, and 0.810 + 0.040), the model trained using FL consistently demonstrates superior AUPRC and accuracy under the conditions of IID (0.323 ± 0.010 and 0.961 ± 0.003) and Real-world (0.311 + 0.011 and 0.970 + 0.002). However, it is noteworthy that in the context of Non-IID, model trained using FL (0.226 + 0.071 and 0.876 + 0.162) lags behind the centralization model.

### Comparing to medical scoring model

**Fig. 5:**
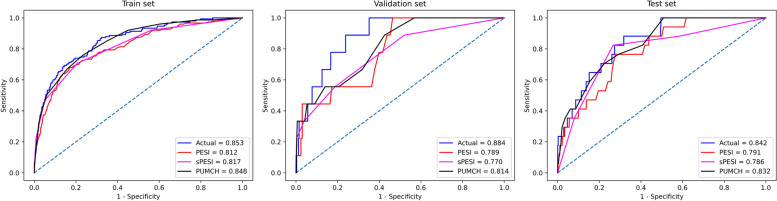
ROC curves on training, validation, and test sets using FL and medical scoring models in Real-world. The blue curve in the figure corresponds to using FL, the red curve corresponds to PESI, the magenta curve corresponds to sPESI and the black curve corresponds to PUMCH

In the experiment, the performance of models that exhibited the best performance in the previous ten trials using FL in the real-world scenario was also compared with PESI, sPESI, and PUMCH models. The results of the experiments are shown in Fig. 5, and the specific results are recorded in Table 4.

**Table 4:** The FL model and medical scoring models in Real-world

Characteristic	Fed	PESI	sPESI	PUMCH
Train set				
AUC	0.853	0.812	0.817	0.848
AUPRC	0.316	0.266	0.277	0.301
Accuracy	0.953	0.474	0.462	0.568
Precision	0.522	0.075	0.076	0.093
Recall	0.107	0.893	0.920	0.920
F1_score	0.179	0.139	0.140	0.168
Validation set				
AUC	0.884	0.789	0.770	0.814
AUPRC	0.216	0.222	0.203	0.254
Accuracy	0.971	0.478	0.490	0.583
Precision	0.333	0.048	0.044	0.053
Recall	0.111	1.0	0.889	0.889
F1_score	0.167	0.091	0.083	0.100
Test set				
AUC	0.842	0.791	0.786	0.832
AUPRC	0.326	0.169	0.102	0.214
Accuracy	0.972	0.493	0.430	0.509
Precision	1.0	0.060	0.051	0.066
Recall	0.176	0.941	0.882	1.0
F1_score	0.300	0.113	0.096	0.123

Fed in the table means model trained using FL in Real-world

*Abbreviations*: *PESI* Pulmonary embolism severity index, *sPESI* Simplified PESI, *PUMCH* Peking Union Medical College Hospital, *AUC* Area under the curve, *AUPRC* Area under the precision-recall curve

On the training, validation and test sets, AUC of model trained using FL (0.853, 0.884 and 0.842) consistently outshines PESI (0.812, 0.789 and 0.791), sPESI (0.817, 0.770 and 0.786) and PUMCH (0.848, 0.814 and 0.832).

From Table 4, it can be seen that on the test set, while the AUPRC, accuracy, precision and F1-score of model trained using FL(0.326, 0.972, 1.0 and 0.300) outperform PESI (0.169, 0.493, 0.060 and 0.113), sPESI (0.102, 0.430, 0.051 and 0.096) and PUMCH (0.214, 0.509, 0.066 and 0.123), its recall (0.176) lags behind PESI (0.941), sPESI (0.882), and PUMCH (1.0).

### Comparing to models trained separately at each client

**Fig. 6:**
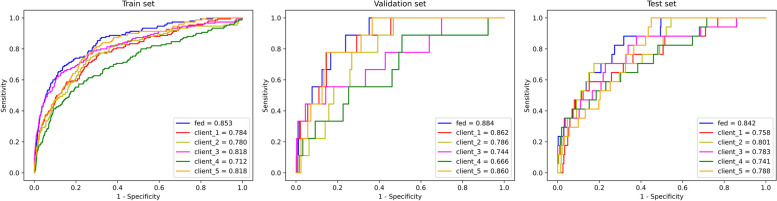
The ROC curves of the model trained using FL (fed) and models independently trained at each individual client on the training, validation and test sets

In this experiment, the best-performing trial among ten real-world experiments was chosen. In this particular trial, the performance of the model trained using FL was compared with models trained individually on five clients. The results are depicted in Fig. 6.

Based on Fig. 6, it's evident that the models trained using FL (0.853, 0.884, 0.842) exhibited superior AUC values across the training, validation, and test sets compared to those trained directly on the clients (0.784, 0.862, 0.758 for client_1, 0.780, 0.786, 0.801 for client_2, 0.818, 0.744, 0.783 for client_3, 0.712, 0.666, 0.741 for client_4, 0.818, 0.860, 0.788 for client_5). Delong tests were employed to evaluate the statistical significance of the performance of the client models compared to FL, which demonstrated the highest AUC, utilizing prediction results from the test set. It was observed that significant differences exist among client_1 (*p* = 0.049 < 0.05), client_2 (*p* = 0.032 < 0.05), and client_5 (*p* = 0.018 < 0.05), while no significant differences were observed between client_3 (*p* = 0.200) and client_4 (*p* = 0.094). However, FL (0.326) exhibited higher AUPRC compared to client_3 (0.289) and client_4 (0.218), indicating its superior performance in balancing precision and recall.

## Discussion

This study is the first to apply FL to prognostic risk assessment of PTE and demonstrate the feasibility of using FL that can combine data from multiple hospitals in a privacy-preserving manner to predict PTE prognostic risk. In reality, FL, a decentralized training strategy, is likely to be a key driving force for the application of artificial intelligence in the field of healthcare, as hospitals may be reluctant to share patient data, and patients are increasingly concerned about preserving their personal privacy. When the amount of corresponding data in hospitals is relatively limited and the labels in corresponding patient data are extremely unbalanced (e.g., only 4.40% of the PTE data in this study are positive), the effectiveness of using FL remains uncertain. The experimental results indicate that the use of FL can improve the generalization performance of the model to some extent, reflecting successful decentralized optimization with diverse distributions of training data.

From the information provided by Fig. 2, the LR model trained using FL can be used to detect the prognostic risk of PTE. In Fig. 2, the predictive performance of LR, CNN, and MLP was compared, and it was found that when the data volume was tiny and the sample labels were extremely imbalanced, sufficient information could not be provided for CNN and MLP to extract meaningful features. This disparity results in the LR (0.842) outperforming both the CNN(0.819) and MLP(0.784) in terms of AUC value.

**Fig. 7:**
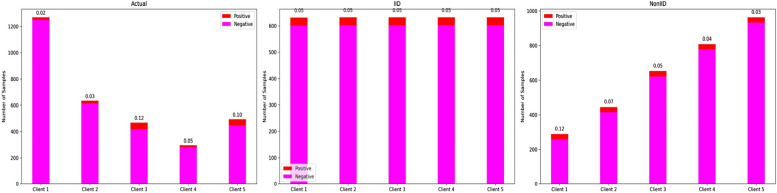
In the bar charts, two components are depicted: the red section represents the number of positive samples for each client, and the magenta section represents the number of negative samples. The sum of these two sections represents the total data volume for each client. The numbers displayed above the bar charts indicate the ratio of positive to negative samples for each client. The three charts, labeled as "Actual," "IID," and "NonIID," respectively, correspond to the data distributions of five clients in the best-performing experiment out of ten simulated experiments under Real-world, IID, and Non-IID

Figure 7 depicted the data distribution among different clients under three scenarios in the experiment: Real-world, IID and Non-IID. It was evident that under Real-world and Non-IID scenarios, the distribution among different clients showed significant discrepancies (both in total data volume and the ratio of positive to negative samples), whereas in the IID scenario, the differences were relatively minor.

In the IID (0.829 ± 0.004; *p* < 0.001) and Real-world, the models trained using FL (0.835 ± 0.005; *p* < 0.001) outperformed centralization model (0.811 ± 0.001) on the test set. This indicated that, in both of the above situations, training models individually at each hospital and then aggregating the results yielded better performance than training a model on the pooled data from all hospitals. In the Non-IID scenario, due to the large differences in data between clients, AUC of the model trained using FL (0.799 ± 0.031) exhibited a marginal decrease compared to centralization model (0.811 ± 0.001) on the test set. This observation indicated that the FedAvg algorithm was not particularly well-suited for scenarios with substantial disparities in client data. However, compared to centralization model, the p-value for the FL model is 0.242. This indicates that even under Non-IID, FL can achieve similar performance to the centralization model.

As illustrated in Table 3, when the model was set to the same classification threshold (0.3), in the context of IID (0.235 ± 0), Non-IID (0.312 ± 0.260) and Real-world (0.176 ± 0.028), the recall of model trained using FL lagged behind that of the centralization mode l(0.606 ± 0.092) on the test set. It may be because when solving the problem of having too few positive samples in the data, model trained using FL may not be able to effectively learn the features of positive samples, resulting in lower predictive performance on positive samples compared to centralization model. However, while centralization model achieved a higher recall, its precision (0.109 ± 0.014) fell below model trained using FL (0.413 ± 0.065 for IID, 0.175 ± 0.120 for Non-IID and 0.792 ± 0.158 for Real-world) on the test set, meaning that many negative samples were predicted as positive samples. If the model trained using FL wants to achieve the same effect, the classification threshold of the model trained using FL needs to be set lower than that of centralization model.

In Fig. 5, the performance of the model trained using FL in the Real-world was also compared with the performance of three medical scoring models: PESI, sPESI, and PUMCH. Table 4 revealed that the model trained using FL(0.842) outperformed PESI (0.791), sPESI (0.786) and PUMCH (0.832) on the test set. At the same time, it was found that the recall of model trained using FL(0.176) lagged behind PESI(0.941), sPESI(0.882), and PUMCH(1.0) on the test set. This indicated that the model trained using FL had poor classification ability for positive samples when there was a significant difference in the number of positive and negative samples. This phenomenon could be attributed to the limited number of positive samples, posing a challenge for the model to learn meaningful features. In the experiment, a strategic approach to enhance the recall rate involves adjusting the model's classification threshold. After reducing the classification threshold of the model, using FL is also a feasible option for deployment in hospital environments, as it (0.842) surpasses PESI (0.791), sPESI (0.786) and PUMCH (0.832) in terms of generalization.

Figure 6 illustrated the comparison between models trained using FL and those trained directly on data from five individual clients. The AUC values across the test set were notably higher for models trained using FL (0.842) compared to those trained directly on the clients' data (0.758, 0.801, 0.783, 0.741, 0.788). This is because in the training process, FL effectively utilizes datasets from all clients, while each client only uses its local dataset to train the model. This underscores the advantage of FL.

**Fig. 8:**
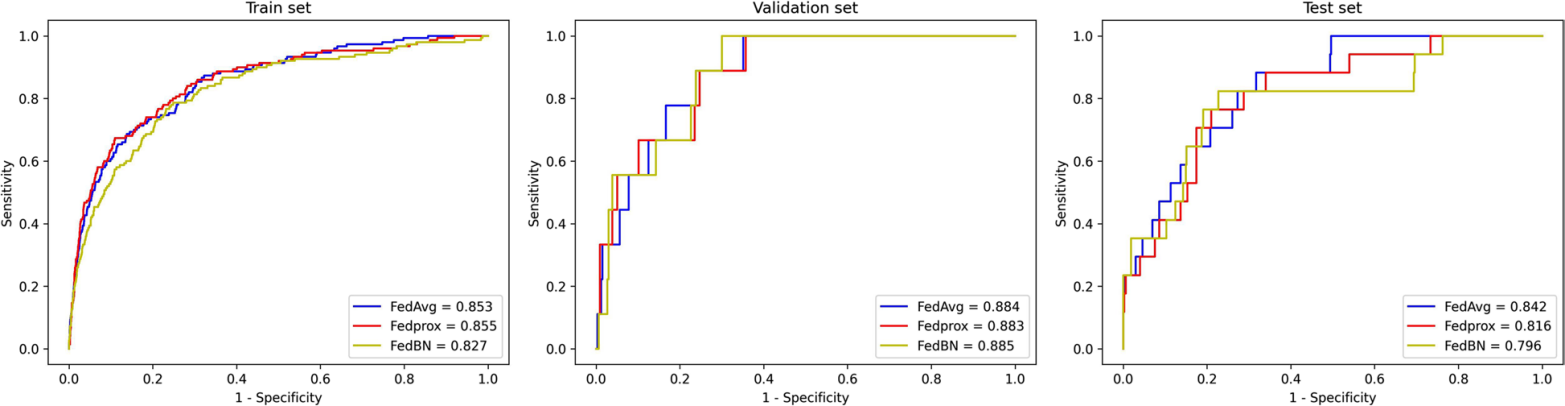
The ROC curves of models trained using FedAvg, Fedprox and FedBN on the training, validation and test sets

In Fig. 8, the performance of the FedAvg algorithm was compared with two optimization algorithms for FL, Fedprox and FedBN, addressing Non-IID data, across the training, validation, and test sets. It was observed that while FedAvg exhibited similar performance to Fedprox [29] and FedBN [30] on the training and validation sets, its generalization performance (0.842) on the test set surpassed that of Fedprox (0.816) and FedBN (0.796). This discrepancy stemmed from the fact that existing FL algorithms designed to handle Non-IID data may not have adequately addressed the specific Non-IID characteristics present in our dataset. Additionally, as the FedAvg algorithm is a classic algorithm in FL, it may have been more robust and generally applicable in certain scenarios.

Although this study proves that there are some advantages of using FL to predict PTE prognostic risk, there are some limitations in this study. In the Non-IID scenario, the performance of the model trained using FL will be degraded, which may be caused by three parts of the factors. First, the amount of data in the hospitals themselves in the experiments is too limited, and there is a large difference in the distribution of the data in each hospital. Second, the number of patients who are positive in the data of the hospitals is slight, and the class imbalance of labels has a certain impact on the experimental results. Third, the FedAvg algorithm employed in the experiments aggregates models based on a simple weighted average of data volumes at each hospital, which doesn't effectively address the disparities in data distributions among different hospitals. In addition, due to limitations of the dataset, only a 30-day follow-up period could be considered in this experiment.

The current study is retrospective, with plans for future prospective, multicenter validation, encompassing a broader patient cohort and incorporating follow-up data at 60/90/180 days. Additionally, since current experiment only utilizes the FedAvg algorithm of FL, the focus will be on designing aggregation algorithms that can improve model performance when the data differences between clients are relatively large. Furthermore, subsequent research can include a larger volume of patient data and a more extensive number of hospitals, enhancing the generalization capabilities of the model trained using FL.

## Conclusions

Based on experimental results on real clinical data from multiple centers, this study demonstrates that FL can be used to construct a prognostic risk prediction model for PTE, and it is suitable for deployment in hospitals, which is helpful for clinical practice.

## Data Availability

Data are available upon reasonable request to the corresponding author.

## Abbreviations

AI: Artificial intelligence
AUC: Area under the receiver operating characteristic curve
AUPRC: Area under the precision-recall curve
BP: Blood pressure
CNN: Convolutional neural network
FL: Federated learning
FedAvg: Federated averaging
Fed: Model trained using federated learning
IID: Independent identically distributed
LR: Logistic regression
MLP: Multi layer perceptron
Non-IID: Non-independent identically distributed
NonFed: Centralization model
PTE: Pulmonary thromboembolism
PESI: Pulmonary embolism severity index
PUMCH: Peking Union Medical College Hospital
ROC: Receiver operating characteristic
sPESI: Simplified pulmonary embolism severity index
